# Associations of Mid‐Life Peripheral Blood‐based Markers of Inflammation and Alzheimer’s Disease and Related Dementias in the Family and Community Health Study

**DOI:** 10.1002/alz70861_108222

**Published:** 2025-12-23

**Authors:** Sarrah E. Ankeny, Mei Ling Ong, Steven R. H. Beach, Man‐Kit Lei, Ronald L. Simons, Michelle M Mielke

**Affiliations:** ^1^ Wake Forest University School of Medicine, Winston‐Salem, NC USA; ^2^ University of Georgia, Athens, GA USA; ^3^ Division of Public Health Sciences, Wake Forest University, School of Medicine, Winston‐Salem, NC USA

## Abstract

**Background:**

Inflammatory processes are associated with aging, neurodegeneration, and cognitive decline and are proposed to have a potential role in the preclinical stage of dementia. However, studies examining the relationship between systemic inflammation and Alzheimer’s disease and related dementias (ADRD) blood‐based biomarkers (BBMs) are mixed. Few studies have examined associations between inflammation and ADRD BBMs in mid‐life, when changes in inflammation related to preclinical dementia pathology would most likely occur. This study examined the cross‐sectional associations between midlife peripheral measures of inflammation and ADRD BBMs in an underserved cohort.

**Method:**

This study included 305 middle‐aged (mean [SD] age, 47.5 [8.6] years) African American participants from the Family and Community Health Study (FACHS). Cross‐sectional associations between serum‐levels of Tumor Necrosis Alpha (TNFα), Interleukin (IL)‐1β, IL‐6, Macrophage Inflammatory Protein (MIP)‐1β, and an epigenetic measure of C‐Reactive Protein (CRP) with serum‐levels of phosphorylated tau 181 (pTau181), neurofilament light (NfL), and glial fibrillary acidic protein (GFAP) were analyzed using linear regression. Prior to analyses, peripheral inflammatory markers and ADRD BBMs were log (log(x+1)) transformed to correct for right‐skewed distributions. Models were adjusted for age, gender, and education.

**Result:**

Serum levels of peripheral inflammation were significantly associated with serum levels of GFAP. A one‐unit increase in TNFα (*β* = ‐0.026, *p* = 0.008), IL1β (*β* = ‐0.031, *p* = 0.009), IL6 (*β* = ‐0.026, *p* = 0.027), MIP1β (*β* = ‐0.041, *p* = 0.027), and CRP methylation (*β* = ‐0.076, *p* = 0.027) were associated with lower GFAP. Serum levels of peripheral inflammation were not significantly associated with serum levels of pTau181 or NfL.

**Conclusion:**

We found negative association between peripheral markers of inflammation and GFAP in this mid‐life underserved cohort. Future analyses will investigate associations between peripheral markers of inflammation and 10‐year change in ADRD BBMs in this cohort.

